# CACN-1/Cactin Plays a Role in Wnt Signaling in *C. elegans*

**DOI:** 10.1371/journal.pone.0101945

**Published:** 2014-07-07

**Authors:** Melissa LaBonty, Cleo Szmygiel, Lauren E. Byrnes, Samantha Hughes, Alison Woollard, Erin J. Cram

**Affiliations:** 1 Department of Biology, Northeastern University, Boston, Massachusetts, United States of America; 2 Department of Biochemistry, University of Oxford, Oxford, United Kingdom; Brown University/Harvard, United States of America

## Abstract

Wnt signaling is tightly regulated during animal development and controls cell proliferation and differentiation. In *C. elegans*, activation of Wnt signaling alters the activity of the TCF/LEF transcription factor, POP-1, through activation of the Wnt/β-catenin or Wnt/β-catenin asymmetry pathways. In this study, we have identified CACN-1 as a potential regulator of POP-1 in *C. elegans* larval development. CACN-1/Cactin is a well-conserved protein of unknown molecular function previously implicated in the regulation of several developmental signaling pathways. Here we have used activation of POPTOP, a POP-1-responsive reporter construct, as a proxy for Wnt signaling. POPTOP requires POP-1 and SYS-1/β-catenin for activation in L4 uterine cells. RNAi depletion experiments show that CACN-1 is needed to prevent excessive activation of POPTOP and for proper levels and/or localization of POP-1. Surprisingly, high POPTOP expression correlates with increased levels of POP-1 in uterine nuclei, suggesting POPTOP may not mirror endogenous gene expression in all respects. Genetic interaction studies suggest that CACN-1 may act partially through LIT-1/NLK to alter POP-1 localization and POPTOP activation. Additionally, CACN-1 is required for proper proliferation of larval seam cells. Depletion of CACN-1 results in a loss of POP-1 asymmetry and reduction of terminal seam cell number, suggesting an adoption of the anterior, differentiated fate by the posterior daughter cells. These findings suggest CACN-1/Cactin modulates Wnt signaling during larval development.

## Introduction

Wnt signaling is crucial for proper cell fate specification, cell migration, and cell division. For example, Wnt signaling drives axial polarization throughout the animal kingdom [1], [2], generates attractive and repulsive cues that guide axon migration [3], and induces the activity of Cyclin D1, a regulator of cell cycle progression [4]. Aberrant Wnt signaling can lead to developmental disorders and metastatic cancer [5]. Wnt signaling encompasses a diverse set of activators and effectors, including regulators of the canonical Wnt/β-catenin pathway, the Wnt/β-catenin asymmetry pathway, and the Wnt/planar cell polarity (PCP) pathway [6], [7]. In *C. elegans*, signaling through the canonical and the non-canonical asymmetry pathways results in stabilization and nuclear localization of β-catenin, thereby providing a partner for the TCF/LEF family of transcription factors, and allowing activation of gene expression [6], [8], [9]. In addition, the asymmetry pathway activation of Nemo-like kinase (NLK) regulates nuclear-cytoplasmic cycling of TCF/LEF [8], [10]. Vertebrates produce a single β-catenin to modulate multiple TCF/LEF transcription factors [11]. The *Drosophila* genome encodes a single β-catenin and a single TCF/LEF transcription factor [12]. In contrast, the *C. elegans* Wnt signaling pathways utilize several divergent β-catenins (SYS-1, WRM-1, and BAR-1) to regulate a single TCF/LEF homolog, POP-1 [13]–[17].

POP-1 is a master regulator of gene expression and cell fate determination [18]–[22]. POP-1 can act to activate or repress genes depending on the cell type, availability of activators and promoter contexts [13], [23]–[26]. For example, in the cell division that produces E and MS daughter cells in the early *C. elegans* embryo, asymmetric localization of POP-1 is required for proper specification of cell fates [19]. In the high POP-1 expressing MS cells, endodermal (E) fates are repressed. Loss of function mutations in *pop-1* lead both daughter cells to adopt E-like fates, indicating the repressive function of POP-1 is required in MS [18], [23], [27], [28]. Some evidence suggests that the lower levels of POP-1 contribute to activation of genes such as *end-1, 3* and *sdz-23, 26* in the E lineage [23], [29]. Recently, differentially regulated POP-1 targets have been identified in the *C. elegans* embryo, which can be repressed (*ceh-36*, *ceh-27*, *tbx-11*, *nhr-25*, *tlp-1* and *elt-6*) or activated (*tlp-1*, *elt-6* and *pha-4*) depending on context [26]. The emerging picture is that POP-1 generally acts a repressor, but in the presence of its activating β-catenin partner, can also activate gene expression.

There is a novel, non-canonical, Wnt pathway in *C. elegans* termed the Wnt β-catenin asymmetry pathway, in which the ratio of nuclear POP-1 to its activating β-catenin is thought to be the primary determinant of whether POP-1 target genes are repressed or activated [13], [15], [30]. Inactive Wnt signaling is correlated with high nuclear POP-1, low levels of SYS-1/β-catenin, and repression of gene expression [22], [23], [27], [29]. Nuclear levels of POP-1 are controlled by regulated nuclear export, in which WRM-1/β-catenin works in conjunction with the Nemo-like kinase LIT-1 [31]. WRM-1 is asymmetrically distributed in dividing cells, being preferentially localized to the nuclei of posterior daughters, and to the cortex of anterior daughters [8], [32]. High levels of nuclear WRM-1 result in the export of POP-1 from the posterior nucleus following phosphorylation of POP-1 by LIT-1 [10], [31]. For example, during *C. elegans* larval development, most seam cells divide asymmetrically to produce two daughter cells; an anterior cell that differentiates and later fuses with the hypodermis and a posterior cell that retains the proliferative ability (the seam cell fate) [8], [19], [20], [33]. This division is controlled by the Wnt/β-catenin asymmetry pathway [34], and in anterior daughters, a high nuclear POP-1 to SYS-1 ratio leads to repression of seam cell fate [19]. The importance of POP-1 in this decision is illustrated by the finding that in *pop-1* RNAi animals, all daughters adopt the seam cell fate, leading to a dramatic increase in the number of seam cells [34].

The reporter TOPFLASH has been used extensively to investigate TCF/LEF regulation of gene expression in mammalian cells [35]–[37]. POPTOP, a similar construct composed of tandem POP-1 binding sites fused to a minimal promoter, has been used in *C. elegans* to investigate activation of transcription by POP-1 in several different cell types [38], [39]. POPTOP is transiently expressed during stages of development that require the activation of POP-1-mediated transcription, including the M cell lineage during the L1-L2 larval stages [40], in VPC progeny during L3 larval stage [38], and in the somatic gonad of the male during the L3 larval stage [41]. Our work focuses on the expression of the POPTOP reporter in the tissues of the developing somatic gonad during the L4 larval stage.

Cactin is a well-conserved, nuclear protein that has been characterized as a negative regulator of many different developmental processes. Cactin seems to play a role in NF-KB/Rel signaling, thereby impacting development and innate immunity in *Drosophila* [42], zebrafish [43] and human cell culture [44]. Cactin also regulates expression of G1 cell cycle genes in the protozoan parasite *T. gondii* [45]. In *Arabidopsis*, CACTIN is required for embryogenesis [46]. Accumulating evidence from studies in various organisms suggests Cactin interacts genetically and biochemically with spliceosomal proteins and may be involved in RNA processing, stability or post-transcriptional gene regulation [46]–[50], which could help explain how Cactin homologs impact these disparate processes. In *C. elegans*, CACN-1/cactin is expressed in tissues of the developing somatic gonad and is required for larval morphogenesis, including proper distal tip cell (DTC) migration and somatic gonad development [51].

In this study we describe a potential novel role for CACN-1 as a regulator of Wnt signaling. We demonstrate that loss of CACN-1 in the developing L4 somatic gonad elicits POP-1 and SYS-1 dependent increases in expression of the reporter POPTOP, suggesting CACN-1 normally functions as an inhibitor of POP-1-dependent transcription. Loss of CACN-1 alters the expression pattern of POP-1 at least in part by regulating the localization of the negative regulator, LIT-1. Contrary to expectation, high levels of POP-1 coincide with high levels of POPTOP expression. This is the opposite of how targets of the Wnt/asymmetry pathway in other cell types typically respond; therefore, we discuss the potential limitations of the POPTOP system for investigating Wnt signaling. Importantly, we demonstrate that CACN-1 is required for robust anterior/posterior asymmetry of POP-1 and normal proliferation of larval seam cells, a process tightly regulated by POP-1 and the Wnt/β-catenin asymmetry pathway.

## Materials and Methods

### Nematode strains

Nematodes were cultivated on NGM agar plates with *E. coli* OP50 bacteria according to standard techniques [52]. Nematode culture and observations were performed at 23°C, unless otherwise indicated. JK3437 expresses GFP::POP-1. The promoter driving this construct *jmp#1* [30] was confirmed to be *sys-1p* by sequencing. The strains used are listed in Table 1.

**Table 1:** C. elegans strains used in this study.

Strain Name	Genotype
AW763	*unc-119(ed4)III; qyIs97; ouEx610*
DZ325	*ezIs2[fkh-6::gfp, unc-119(+)] III; him-8(e1489) IV*
EU603	*lit-1(or131) III; him-8(e1489) IV*
JK2945	*pop-1(q624) I/hT2[bli-4(e937) let-?(q782) qIs48] (I;III)*
JK3437	*him-5(e1490) V; qIs74[Psys-1::gfp-pop-1]*
JR667	*unc-119(e2498)III; wIs51[pMF1+pDP#MM016β] V*
N2	Bristol N2, Wildtype
NH2246	*ayIs4[egl-17p::gfp; dpy-20(+)] I; dpy-20(e1282) IV*
NK598	*unc-119(ed4)*; qyIs96[*5300cacn-1*::GFP, *unc-119*(+)]
PS5332	*unc-119(ed4) III; him-5(e1490) V; syIs187[pes-10::7XTCF-mCherry-let-858(3′UTR); unc-119(+)]*
UN1119	*syIs187[pes-10::7XTCF-mCherry-let-858(3′UTR); unc-119(+)]; pop-1(q624) I*
UN1156	*syIs187[pes-10::7XTCF-mCherry-let-858(3′UTR); unc-119(+)]; lit-1(or131) III*
UN1157	*syIs187[pes-10::7XTCF-mCherry-let-858(3′UTR); unc-119(+)]; lit-1(ne1991) III*
UN1208	*syIs187[pes-10::7XTCF-mCherry-let-858(3′UTR); unc-119(+)]; ezIs2[fkh-6::gfp, unc-119(+)] III*
UN1210	*syIs187[pes-10::7XTCF-mCherry-let-858(3′UTR); unc-119(+)]; ayIs4[egl-17p::gfp; dpy-20(+)] I; dpy-20(e1282) IV*
WM79	*rol-6(n1270) II; neEx1[lit-1::gfp]*
WM93	*lit-1(ne1991) III*

### Genetic Crosses

The lines UN1119, UN1156, UN1157, UN1208, and UN1210 were each generated by crossing POPTOP males with hermaphrodites containing *pop-1(q624)*, *lit-1(or131)*, *lit-1(ne1991)*, *ezIs2*, *ayIs4*, or *sEx10874*, respectively. Homozygosity of alleles or transgenes was confirmed by PCR genotyping of the progeny and by observation of reporter expression using GFP or mCherry epifluorescence microscopy.

### Generation of transgenic animals

*NK598* animals (a gift from David Sherwood, Duke University, Durham, NC) carry an integrated transcriptional fusion of *cacn-1*::GFP including 5.3 kb of sequence upstream of the W03H9.4 translational start site. We used an *ajm-1*::mCherry reporter to outline the seam cells allowing us to observe *cacn-1*::GFP expression in these cells. Plasmid p*AW525* (*ajm-1*::mCherry) was injected into the syncytial gonad of *NK598* L4 hermaphrodite animals (at a concentration of 10–20 ng/µl) generating strain *AW763*.

### RNAi

Starved nematodes were allowed to recover on fresh OP50 seeded NGM plates for two days at 23°C. Temperature sensitive strains *lit-1(or131)*; POPTOP and *lit-1(ne1991)*; POPTOP were recovered for four days at a permissive temperature of 15°C. This procedure produces gravid young adult hermaphrodites for egg collection. Eggs were released using alkaline hypochlorite solution [53]. Following two washes in M9 buffer, eggs were transferred to plates seeded with RNAi HT115(DE3) bacteria expressing dsRNA. The nematode strain used in each experiment is indicated.

The RNAi feeding protocol was essentially as described [54]. Bacteria were cultured overnight in LB supplemented with 40 µg/ml ampicillin and seeded onto NGM agar supplemented with carbenicillin (25 µg/ml) and IPTG (1 mM). dsRNA expression was induced overnight at room temperature on the IPTG plates. Eggs were then transferred onto the plates and the RNAi phenotypes were monitored at age-matched time points. RNAi experiments were performed at 23°C, except for experiments with strains *lit-1(or131)*; POPTOP and *lit-1(ne1991)*; POPTOP, which required growth at the permissive temperature of 15°C for 48 hours, followed by growth at the non-permissive temperature of 23°C for 24 hours. Because the sterility and embryonic lethality caused by *cacn-1* RNAi normally preclude scoring F1 animals, attenuated RNAi was used for analysis of the seam cell divisions. The genomic *cacn-1* RNAi clone from the Ahringer library, in our hands, is somewhat less effective than the full-length *cacn-1* cDNA clone used for most other experiments (details below). Both RNAi constructs yield similar POPTOP and developmental phenotypes, however, the lower levels of embryonic lethality produced by the Ahringer construct allow analysis of seam cell phenotypes in F1 *scm-1p*::GFP animals. Animals were partially synchronized, reared to L4 on OP50, and then transferred to *cacn-1* RNAi. Surviving F1 progeny were scored at L4, after the final seam cell division, but prior to terminal fusion with the hypodermis, for seam cell number.

The *cacn-1* ORF RNAi clone is a full-length cDNA matching WormBase (WS200) predictions (Open Biosystems; Huntsville, AL, USA). The 5′ *cacn-1* RNAi clone is a 425 bp fragment of *cacn-1* generated by RT-PCR amplification of N2 total RNA and cloned into the RNAi feeding vector *pPD129.36* (Fire Vector Kit). The 3′ *cacn-1* RNAi clone is a genomic clone from well II9E09 of the Ahringer RNAi library [55]. *sys-1* (592 bp fragment), *bar-1* (883 bp fragment), *wrm-1* (510 bp fragment), and *hmp-2* (496 bp fragment) were all cloned from N2 cDNA into the RNAi feeding vector *pPD129.36*. The *lit-1* RNAi clone is a full-length cDNA clone from the ORFeome RNAi library (Open Biosystems; Huntsville, AL, USA). To generate the *sys-1+cacn-1* double RNAi clone, *pUN298*, 986 bp of *sys-1* was amplified from N2 cDNA and cloned into *pUN297* (*cacn-1* in *pPD129.36*) using *Pst*I and *Xho*I sites downstream of the *cacn-1* insert. This process was repeated with *pop-1* specific primers to generate the *pop-1+cacn-1* double RNAi clone, *pUN300*. *sys-1* and *pop-1* were also placed in *pPD129.36* alone using *Pst*I and *Xho*I (*pUN299* and *pUN301*, respectively) and were used as controls in the double RNAi knockdown experiments. Control *pPD129.36* vector was used as a negative control in all RNAi experiments. All primer sequences and cloning details are available upon request.

### Fluorescence Microscopy

To view fluorescent reporter expression patterns, age-matched animals were mounted in a drop of M9 containing 0.08 M sodium azide on a slide coated with 2% agarose in water and imaged using a 60× oil-immersion objective with a Nikon Eclipse 80i epifluorescence microscope equipped with a SPOT RT3 CCD camera (Diagnostic Instruments; Sterling Heights, MI, USA) or Zeiss AxioSKOP2 microscope with a Zeiss AxioCamMR digital camera. Images were captured using either SPOT Advanced version 4.6.4.6 software (Diagnostic Instruments; Sterling Heights, MI, USA or Axiovision software (Release 4.5). Confocal images were taken on a Leica TCS SP5II using Leica Application Suite Advanced Fluorescence Lite software (Release 2.2.1). Animals were mounted on agarose pads (2% agarose, 0.5% 1-phenoxy-2-propanol in M9) in 0.2% 1-phenoxy-2-propanol. The mCherry fluorescence in POPTOP-containing strains was imaged with a 120 ms exposure, with all other microscope settings remaining constant to allow for comparison of different treatments. The individual images were cropped to 500×1000 pixels and mean pixel intensity (arbitrary units, a.u.) was calculated by subtracting the background and then dividing total pixel intensity by total image area using ImageJ. Means for different treatments were compared with an unpaired t-test using GraphPad Prism statistical software. The total number of POPTOP expressing nuclei was determined from maximum intensity projections generated with ImageJ of image stacks through the z-plane of the animals. Nuclear numbers and intensities were scored blindly and means for different treatments were compared with an unpaired t test using GraphPad Prism statistical software. The numbers of seam cell pairs were counted for the various treatments and were compared with an unpaired t-test using GraphPad Prism statistical software. Seam cell GFP fluorescence in JK3437 (*sys-1p*::GFP::POP-1) was calculated by defining a region of interest (ROI) enclosing each seam cell nucleus, quantifying the total pixel intensity of each ROI, and subtracting the background using ImageJ. The area of the ROI was held constant for each seam cell pair. The posterior/anterior ratio was then calculated and means were compared using an unpaired t-test with the GraphPad Prism statistical software.

### Western Blotting

Protein lysates were prepared from age-matched animals grown on *E. coli* HT115(DE3) carrying control RNAi vector or *cacn-1* RNAi vector as follows: Animals were washed from plates using M9 with 0.1% Tween and allowed to settle. After washing, worm cuticles were disrupted by chopping with a razor blade. Animals were resuspended in cold lysis buffer (10 mM Tris pH 8.8, 150 mM NaCl, 0.5 mM EDTA, 0.05% NP40) containing protease inhibitors (Halt Protease Inhibitor Cocktail, Thermo Scientific; Rockford, IL, USA). Protein extracts were obtained by Dounce homogenization for 50–100 strokes on ice. Lysates were clarified by centrifuging for 5 minutes at 20,000 *g*. The protein in the supernatant was quantified using the Precision Red Protein Assay Reagent (Cytoskeleton; Denver, CO, USA). Samples were loaded onto Mini-PROTEAN TGX precast 10% polyacrylamide gels (Bio-Rad; Hercules, CA, USA) for SDS-PAGE. Antibodies used for Western blotting are anti-POP-1 at 1∶1000 (94I, gift from R. Lin), anti-GFP at 1∶1000 (G1544, Sigma), and anti-Actin at 1∶2000 (MAB1501, Millipore). Secondary antibodies used were goat anti-rabbit (PI31460, Thermo Scientific Pierce) and donkey anti-mouse (SA1100, Thermo Scientific Pierce).

## Results

### POPTOP is expressed in the somatic gonad during L4

**Figure 1:**
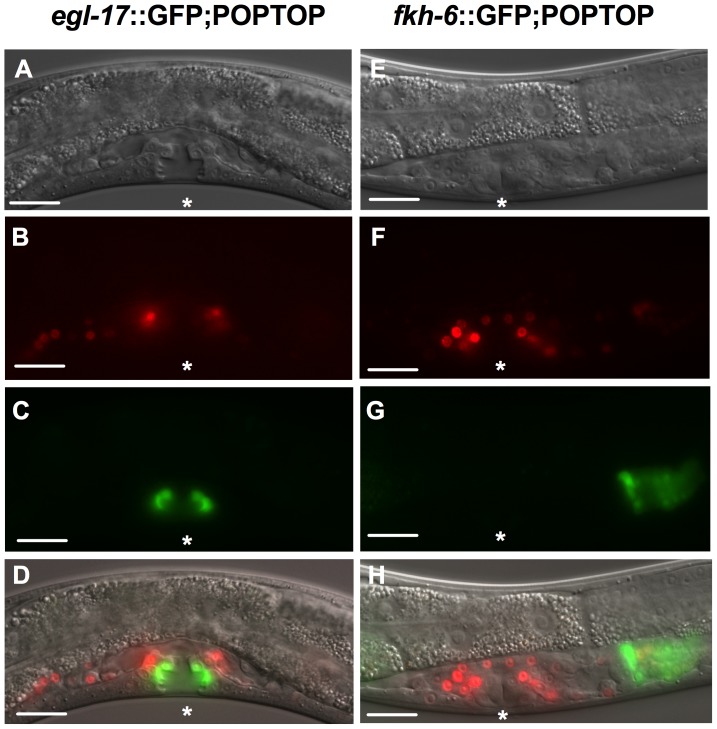
POPTOP is expressed in the L4 somatic gonad. GFP and POPTOP expression are shown in (A–D) *egl-17*::GFP; POPTOP expressing animals or (E–H) *fkh-6*::GFP; POPTOP expressing animals. POPTOP co-localizes with *fkh-6*::GFP in the L4 spermatheca and in cells surrounding the vulva. Asterisks indicate the position of the vulva. Scale bar is 25 µm.

POPTOP, which consists of seven POP-1 binding sites and the *pes-10* minimal promoter driving the expression of mCherry, has been used in *C. elegans* as a readout for Wnt signal transduction in various tissues throughout larval development [38]. However, POPTOP expression has not been well characterized in L4 hermaphrodites. In order to identify the cell lineages expressing POPTOP at this developmental stage, we generated animals co-expressing POPTOP and other cell-type specific reporters. To determine if POPTOP is expressed in L4 vulval cells, we crossed the POPTOP reporter into a strain that expresses *egl-17*::GFP in the primary and secondary vulval cell lineages during the L4 larval stage [56]. EGL-17 is required in this subset of cells to coordinate the positioning of the migrating sex myoblasts [57]. In this strain, no overlap between the POPTOP and *egl-17*::GFP reporters was observed (Fig. 1A–D), suggesting POPTOP is not strongly expressed in vulval lineage cells at this time point. In order to determine whether POPTOP is expressed in the developing and adult spermatheca, we constructed a transgenic line expressing POPTOP and the transgene *fkh-6*::GFP, which is expressed in the L4 and adult spermatheca and less strongly in the uterus [58]. FKH-6 is a transcription factor required in the spermatheca for terminal cell differentiation [59]. We observed co-expression of *fkh-6*::GFP and POPTOP in the spermatheca (Fig. 1E–H) of late L4 animals, confirming that POPTOP is expressed in the L4 somatic gonad.

### CACN-1 negatively regulates POPTOP expression in the somatic gonad

**Figure 2:**
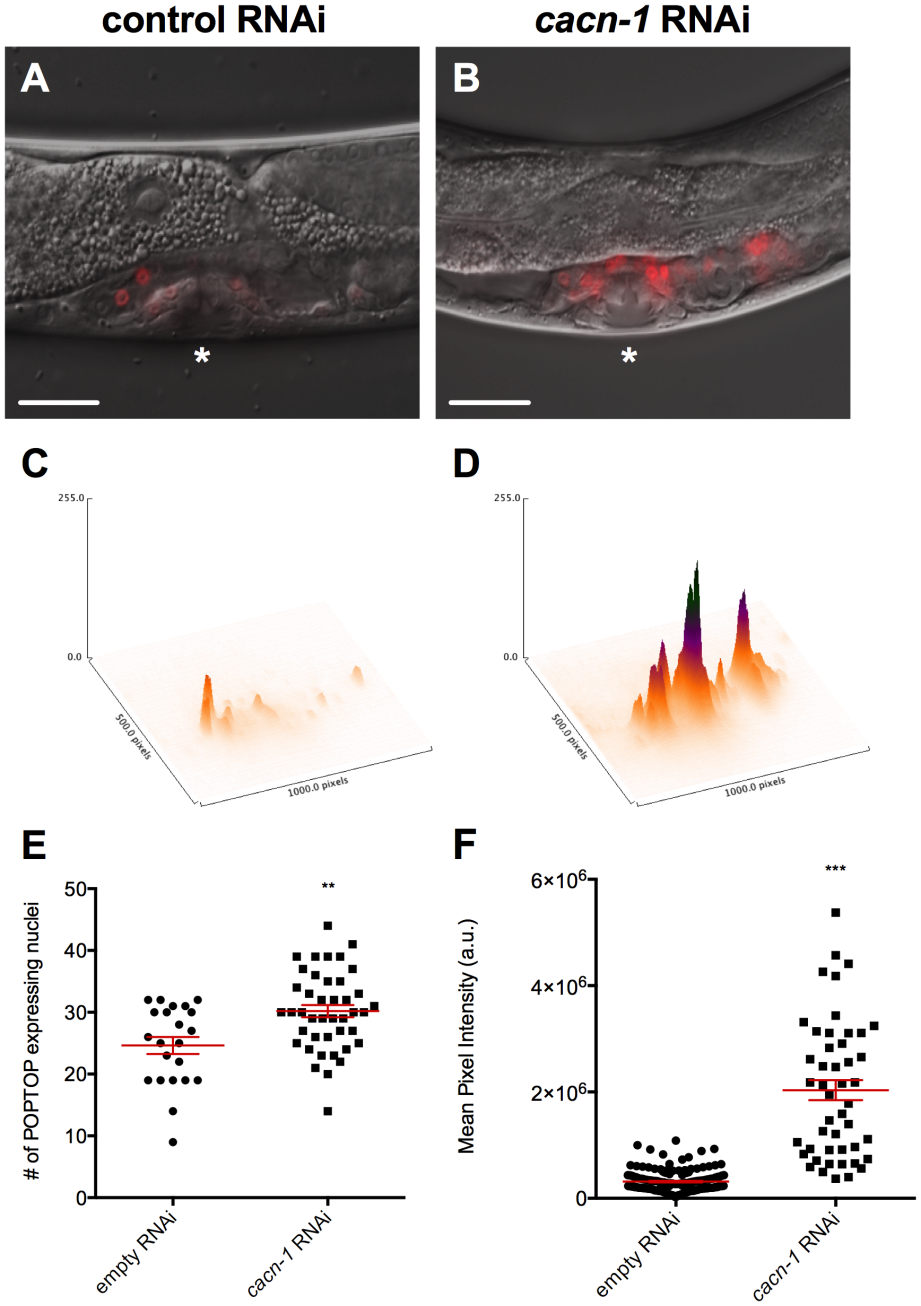
CACN-1 is a negative regulator of POPTOP reporter expression. POPTOP animals were grown on *E. coli* HT115(DE3) carrying the (A) control RNAi vector or (B) *cacn-1* RNAi vector. Asterisks indicate the L4 developing vulva. 3-dimensional representations of POPTOP expression with peak height corresponding to maximum intensity of fluorescent reporter in (C) control RNAi or (D) *cacn-1* RNAi treated animals. (E) Individual points represent the number of POPTOP expressing nuclei for a single animal for a given RNAi treatment. (F) Individual points represent mean pixel intensity (a.u.) per pixel for a single animal for a given RNAi treatment. Error bars indicate mean ± standard error of the mean. *** indicates statistical significance of P<0.001. ** indicates statistical significance of P<0.005. Scale bar is 25 µm.

CACN-1 is expressed in all of the tissues of the developing somatic gonad of L4 animals [51]. In order to assess the effect of loss of CACN-1 on POPTOP expression in these tissues, animals containing the POPTOP reporter were treated with control or *cacn-1* RNAi. POPTOP expression was first quantified in animals treated with negative control RNAi. In these animals, POPTOP expression is limited to the nuclei of a subset of cells in the developing uterus and spermatheca (*n* = 161; Fig. 2A, C). Treatment of POPTOP-containing animals with *cacn-1* RNAi results in an almost 7-fold increase in reporter expression (P<0.0001, *n* = 47; Fig. 2B, D, F). Three different *cacn-1* RNAi constructs (3′, 5′ and ORF) all produced significant changes in POPTOP reporter expression when compared to control treatment (P<0.0001), suggesting the effect is specific to the loss of *cacn-1*. In addition to the enhancement of expression intensity, the number of cell nuclei expressing the POPTOP reporter also increased significantly with *cacn-1* RNAi treatment (P = 0.0016; Fig. 2E). These results suggest CACN-1 may be a negative regulator of POPTOP expression in the somatic gonad.

**Figure 3:**
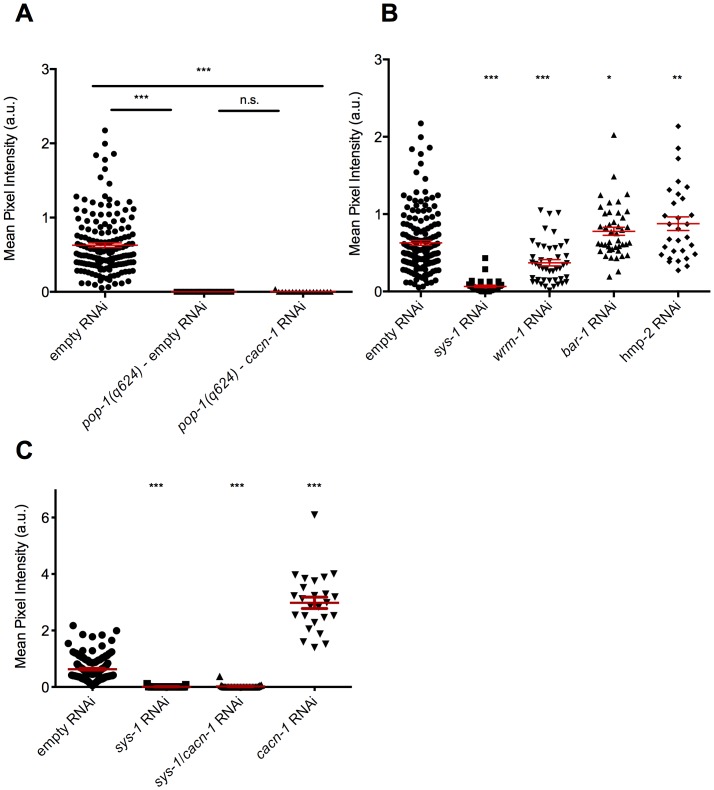
POPTOP expression requires POP-1 and SYS-1. (A) POPTOP or *pop-1(q624)*; POPTOP animals were grown on *E. coli* HT115(DE3) carrying control RNAi or *cacn-1* RNAi vector. (B) POPTOP animals were grown on *E. coli* HT115(DE3) carrying control RNAi, *sys*-1 RNAi, *wrm-1* RNAi, *bar*-1 RNAi, or *hmp-2* RNAi vector. (C) POPTOP animals were grown on *E. coli* HT115(DE3) carrying *cacn-1*+*sys-1* RNAi, *cacn-1* RNAi or *sys-1* RNAi. Individual points represent mean pixel intensity (a.u.) per pixel for a single animal for a given RNAi treatment. Error bars indicate mean ± standard error of the mean. *** indicates statistical significance of P<0.0001, * indicates statistical significance of P<0.05. n.s. is not significant.

To determine whether the effect of *cacn-1* RNAi on POPTOP reporter expression depends on the presence of functional POP-1, we generated animals that expressed both the POPTOP reporter and a partial loss of function allele *pop-1*(*q624*). Our data are consistent with previous results [41] showing that POPTOP expression is dependent on POP-1 (P<0.0001, *n* = 16; Fig. 3A). Importantly, POPTOP reporter expression did not increase in *pop-1(q624)* animals treated with *cacn-1* RNAi (P = 0.3364, *n* = 17; Fig. 3A). These results show that, as expected, control and *cacn-1* RNAi stimulated levels of POPTOP are dependent on functional POP-1.

Based on previous results in other cell types [38], we expected that POP-1 would require a β-catenin partner to activate POPTOP expression. Therefore, we determined the effect of depletion of SYS-1, BAR-1, WRM-1 and HMP-2/β-catenins on POPTOP expression (Fig. 3B). SYS-1 and BAR-1 bind POP-1 in Wnt activated nuclei and are required for activation of transcription by POP-1 in other cell types [16], [60], [61]. WRM-1 binds POP-1, and acts as an adaptor for LIT-1/NLK. Phosphorylation of POP-1 by LIT-1 leads to POP-1 nuclear export [61]. In contrast, HMP-2 does not bind to POP-1 but remains in adherens junctions bound to HMP-1/alpha-catenin and HMR-1/cadherin [60], [62]. Animals treated with *sys-1* RNAi showed a 10-fold decrease in POPTOP reporter expression compared to negative control animals (P<0.0001, *n* = 33; Fig. 3B), suggesting that SYS-1 acts as an important positive regulator of POP-1 in the L4 somatic gonad. None of the other β-catenins tested had a strong effect on POPTOP expression. A modest increase in the average POPTOP signal was observed in the BAR-1 (P = 0.03, *n* = 46; Fig. 3B) and HMP-2 (P = 0.0026 *n* = 30; Fig. 3B) experiments. However, given the low and variable levels of POPTOP expression in control animals, and the established functions of BAR-1 and HMP-2, we suspect these results may not be biologically relevant. Depletion of WRM-1 leads to a slight decrease in POPTOP expression compared to controls (P<0.0001, *n* = 44; Fig. 3B), possibly due to an increase in repressive POP-1 complexes in the nucleus.

Next, we investigated whether SYS-1 was required for the increased POPTOP signal observed in *cacn-1* RNAi treated animals. Because the *sys-1* null phenotype is embryonic lethality [15], and weak *sys-1* mutants are defective in early somatic gonad development [21], we used double RNAi to deplete *sys-1* and *cacn-1* in POPTOP expressing animals. Double RNAi was performed using a single RNAi clone including both *cacn-1* and *sys-1* sequences, and compared to control single RNAi experiments. As expected, *sys-1* RNAi resulted in a significant decrease (P<0.0001, *n* = 29; Fig. 3C), while the *cacn-1* RNAi resulted in an increase in POPTOP expression (P<0.0001, *n* = 29; Fig. 3C) when compared to the negative control. Depleting both *sys-1* and *cacn-1* resulted in a significant decrease in POPTOP expression when compared to the *cacn-1* RNAi (P<0.0001, *n* = 27; Fig. 3C) and negative control (P<0.0001, *n* = 25; Fig. 3C). Taken together, these findings suggest that the activation of POPTOP expression in the *cacn-1* RNAi animals is primarily dependent on SYS-1.

### Loss of CACN-1 results in POP-1 redistribution

**Figure 4:**
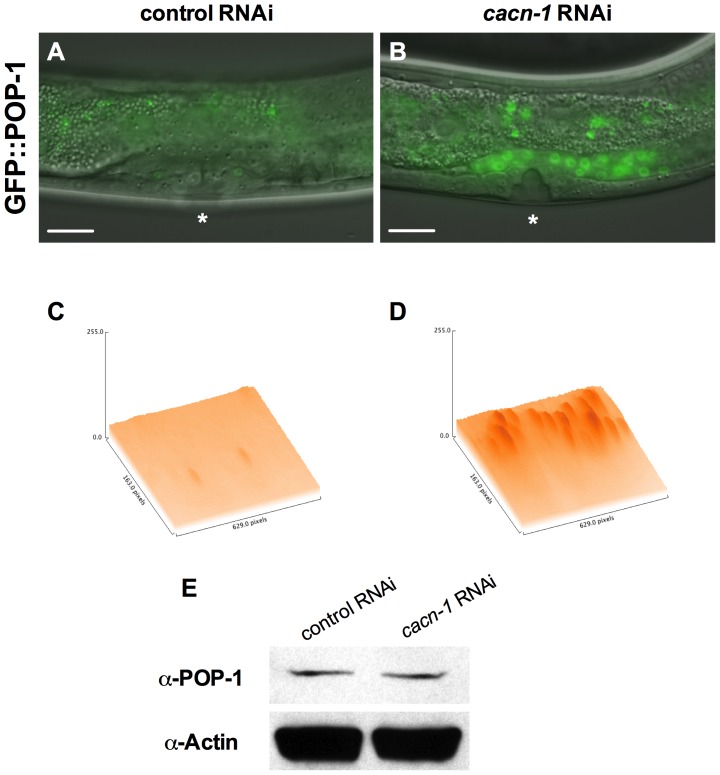
Loss of CACN-1 leads to nuclear accumulation of GFP::POP-1. *sys-1p::*GFP::POP-1 animals were grown on *E. coli* HT115(DE3) carrying (A) control RNAi or (B) *cacn-1* RNAi vector. Asterisks indicate L4 developing vulva. 3-dimensional representations of GFP expression with peak height corresponding to maximum intensity of fluorescent reporter in the uterus and vulva of (C) control RNAi or (D) *cacn-1* RNAi treated animals (E) Western blots of extracts from control RNAi or *cacn-1* RNAi-treated L4 animals probed with anti-POP-1 (94I) and anti-Actin antibodies. Scale bar is 25 µm.

In order to test the hypothesis that CACN-1 might be regulating POPTOP by altering the expression of POP-1, we examined the effect of *cacn-1* RNAi on *sys-1p*::GFP::POP-1. Animals treated with control RNAi show very faint GFP::POP-1 expression in the tissues of the developing somatic gonad during the L4 stage (Fig. 4A, C). Treatment with *cacn-1* RNAi results in increased GFP intensity in the nuclei of the developing uterus and spermatheca (Fig. 4B, D). This change in expression suggests that loss of CACN-1 results in an increase in POP-1 nuclear localization in the cells of the developing somatic gonad. This result is surprising, because in the standard paradigm an increase in POP-1, when accompanied by an increase in the POP-1/SYS-1 ratio, leads to repression of Wnt/asymmetry pathway regulated genes. In contrast, we see increased POP-1 coincident with increased POPTOP expression in the uterus and spermatheca. To assess the overall effect of *cacn-1* RNAi on POP-1 protein levels, we measured POP-1 protein levels in these GFP::POP-1 expressing L4 animals treated with *cacn-1* RNAi (Fig. 4E). Loss of CACN-1 did not cause a significant change in the overall POP-1 protein levels (control RNAi to *cacn-1* RNAi ratio: 0.90, *n* = 2). However, it may not be possible to detect changes in POP-1 protein levels specifically in the uterine and spermathecal cells in the context of whole-animal protein lysates. These results suggest an important function of CACN-1 is to prevent accumulation of POP-1 in the nucleus, perhaps through stimulating export of POP-1 or modulating POP-1 expression. These results also raise a potential concern about the degree to which the POPTOP construct accurately reflects the activation state of endogenous Wnt/asymmetry pathway target genes.

### LIT-1 and CACN-1 both negatively regulate POPTOP expression

**Figure 5:**
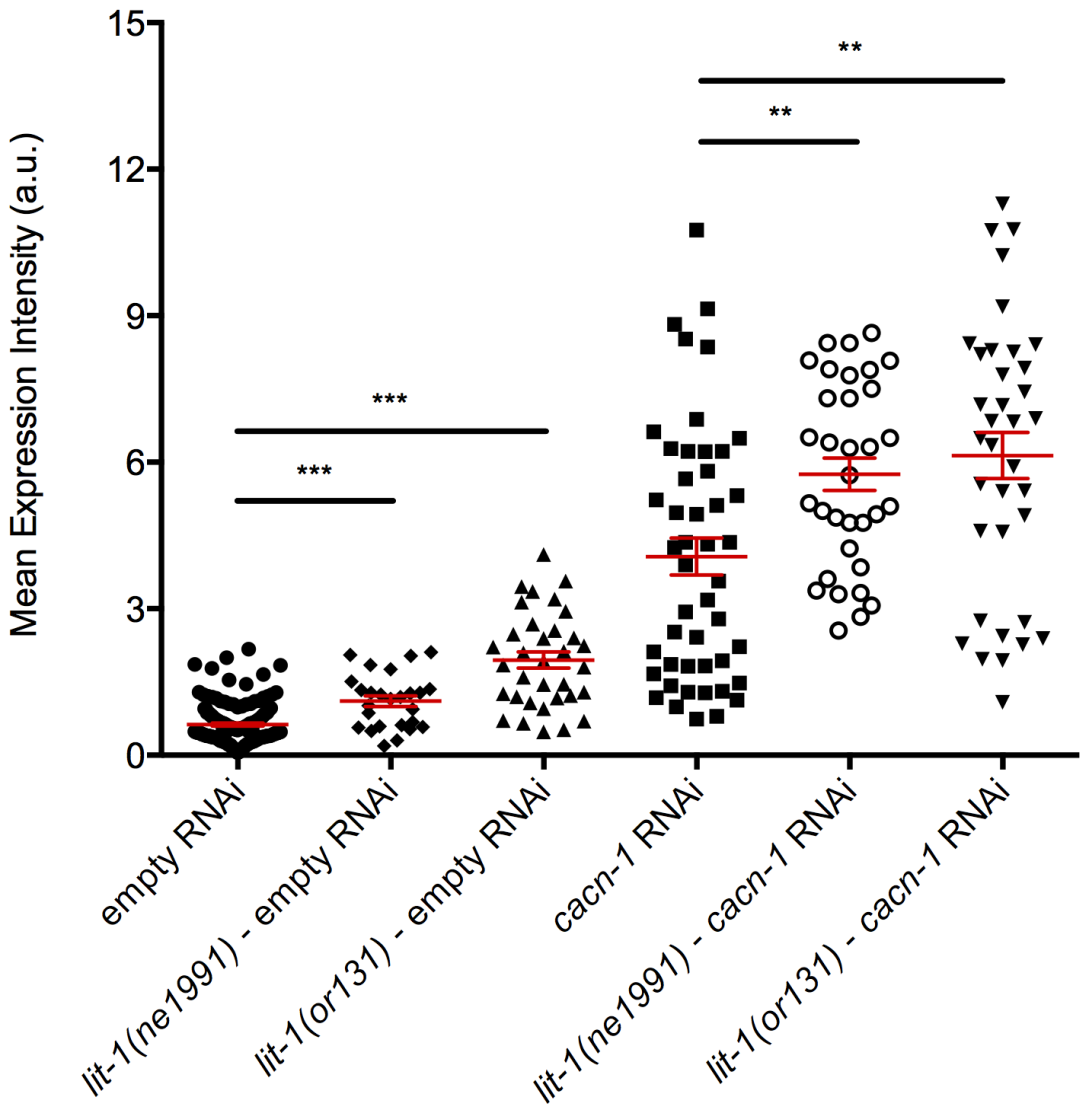
LIT-1 and CACN-1 both negatively regulate POPTOP reporter activity. POPTOP, *lit-1(or131)*; POPTOP, or *lit-1(ne1991)*; POPTOP animals were grown on *E. coli* HT115(DE3) carrying control RNAi or *cacn-1* RNAi. Individual points represent mean pixel intensity (a.u.) per pixel for a single animal for a given RNAi treatment. Error bars indicate mean ± standard error of the mean. *** indicates statistical significance of P<0.0001, ** indicates statistical significance of P<0.005.

The observed nuclear accumulation of POP-1 in CACN-1 depleted animals suggests a possible connection to LIT-1, the *C. elegans* Nemo-like kinase. LIT-1 phosphorylates POP-1 and marks it for export from the nucleus. To determine the effect of loss of LIT-1 on POPTOP expression, we introduced temperature sensitive *lit-1* (*ne1991* and *or131*) alleles into POPTOP-expressing animals. When shifted to the non-permissive temperature of 23°C, the *lit-1* alleles resulted in a two-fold (*lit-1*(*ne1991*); POPTOP P<0.0001, *n* = 26) or three-fold (*lit-1(or131*); POPTOP P<0.0001, *n* = 34) increase in POPTOP reporter expression (Fig. 5). Normally, export of POP-1 results in a decrease in the nuclear POP-1 to SYS-1 ratio and activation of Wnt/asymmetry pathway target genes [10], [63]. Therefore, in the absence of functional LIT-1, POP-1 export should be reduced, resulting in an increase in nuclear POP-1 and a decrease in target gene expression. Although inconsistent with this paradigm, the observed increase in POPTOP expression in *lit-1(ts)* animals is consistent with our previous results suggesting a correlation between increased nuclear POP-1 and POPTOP expression levels in the somatic gonad.

To assess whether CACN-1 works through LIT-1 to exert its inhibitory effects on POPTOP expression, we treated each of the *lit-1(ts)*; POPTOP strains with *cacn-1* RNAi. While RNAi knockdown of CACN-1 alone caused a 7-fold increase in POPTOP reporter expression from wild type levels, *cacn-1* RNAi in combination with the temperature sensitive alleles augmented expression of the POPTOP reporter 10-fold compared to wild type levels (*lit-1*(*ne1991*) P<0.0001, *n* = 33; *lit-1(or131)* P<0.0001, *n* = 36; Fig. 5). The increase in POPTOP reporter expression induced by knockdown of both CACN-1 and LIT-1 protein was also significantly greater than the enhancement by knockdown of CACN-1 alone (*lit-1*(*ne1991*) P = 0.0021; *lit-1(or131)* P = 0.0009). These results suggest that the two proteins may act together or in parallel to negatively regulate POPTOP expression.

### Loss of CACN-1 reduces LIT-1 expression levels

**Figure 6:**
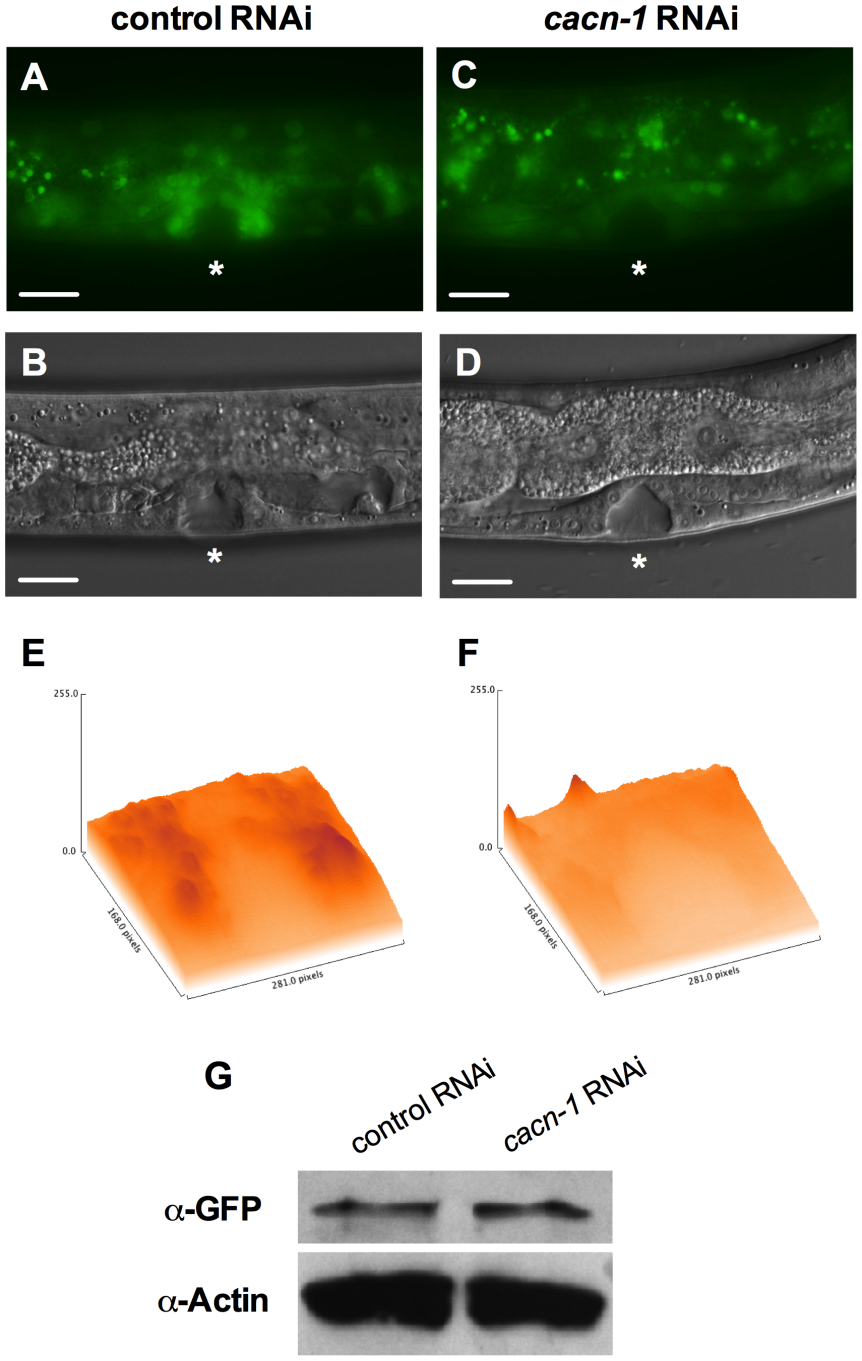
*cacn-1* RNAi reduces LIT-1::GFP expression. LIT-1::GFP animals were grown on *E. coli* HT115(DE3) carrying (A, B) control RNAi or (C, D) *cacn-1* RNAi vector. Asterisks indicate L4 developing vulva. 3-dimensional representations of GFP expression with peak height corresponding to maximum intensity of fluorescent reporter in the uterus and vulva of (E) control RNAi or (F) *cacn-1* RNAi treated animals. (G) Western blots of extracts from control RNAi or *cacn-1* RNAi-treated LIT-1::GFP animals were probed with anti-GFP (LIT-1::GFP) and anti-Actin antibodies. Scale bar is 25 µm.

To further investigate a possible interaction between CACN-1 and LIT-1, we next determined the effect of *cacn-1* RNAi on LIT-1 expression pattern and protein level. LIT-1::GFP is expressed in the developing tissues of the vulva, uterus, and spermatheca (Fig. 6A, B, E). In *cacn-1* RNAi treated animals, uterine and spermathecal LIT-1::GFP expression was reduced (Fig. 6C, D, F). This change in expression indicates that CACN-1 is required for proper expression of LIT-1 in these developing tissues. Although overall LIT-1::GFP protein levels are not reduced (control RNAi to *cacn-1* RNAi ratio: 0.95, *n* = 2; Fig. 6G), it is possible that levels of LIT-1 in somatic gonad and vulval cells are affected by loss of CACN-1. The observed loss of LIT-1 in *cacn-1* RNAi treated animals suggests LIT-1 may also be regulated by CACN-1. These results suggest the loss of both CACN-1 and LIT-1 contributes to the increase in POPTOP expression observed in *cacn-1* RNAi treated animals.

### CACN-1 regulates POP-1 localization in asymmetrical seam cell divisions

So far, our results suggest that CACN-1 may play a role in the Wnt/asymmetry pathway by regulating levels and/or localization of POP-1 and LIT-1. However, many of our conclusions are based on analysis of the POPTOP reporter, the behavior of which is in some respects inconsistent with the expected behavior of endogenous Wnt/asymmetry pathway target genes. Ideally, we would investigate the role CACN-1 plays in regulation of endogenous POP-1 target genes. However, POP-1 target genes have not been identified in the L4 uterus or spermatheca. Therefore, we turned instead to the well-characterized seam cell system to investigate a possible role for CACN-1 in Wnt signaling.

**Figure 7:**
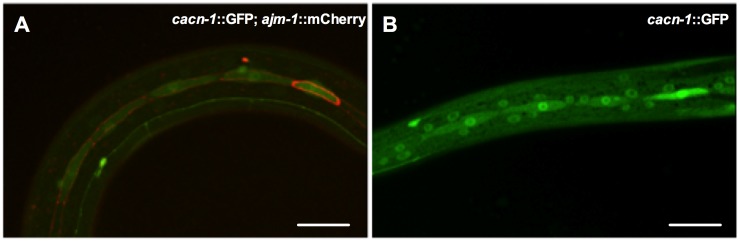
CACN-1 is localized to seam cells. (A) CACN-1 expression is shown in seam cells of *cacn-1*::GFP; *ajm-1*::mCherry animals. (B) *cacn-1*::GFP expression in seam cells and differentiated hypodermal cells. Scale bar is 20 µm.

An important function of POP-1 and the Wnt/β-catenin asymmetry pathway is to regulate the asymmetric division of the larval seam cells [19], [34], [64]. Importantly, CACN-1 is expressed in seam cells (Fig. 7A, B). Elevated WRM-1/β-catenin in the posterior daughter nucleus of an asymmetrically dividing cell results in the export of POP-1 [10], [31], [66]. A lowered POP-1/SYS-1 ratio leads to the transcriptional activation of target genes in the posterior daughter cell. In the anterior daughter cell, high nuclear POP-1 levels lead to the repression of target genes [8], [32], [66]. Therefore, asymmetric distribution of POP-1 in the daughter cells ultimately establishes the appropriate developmental fate.

**Figure 8:**
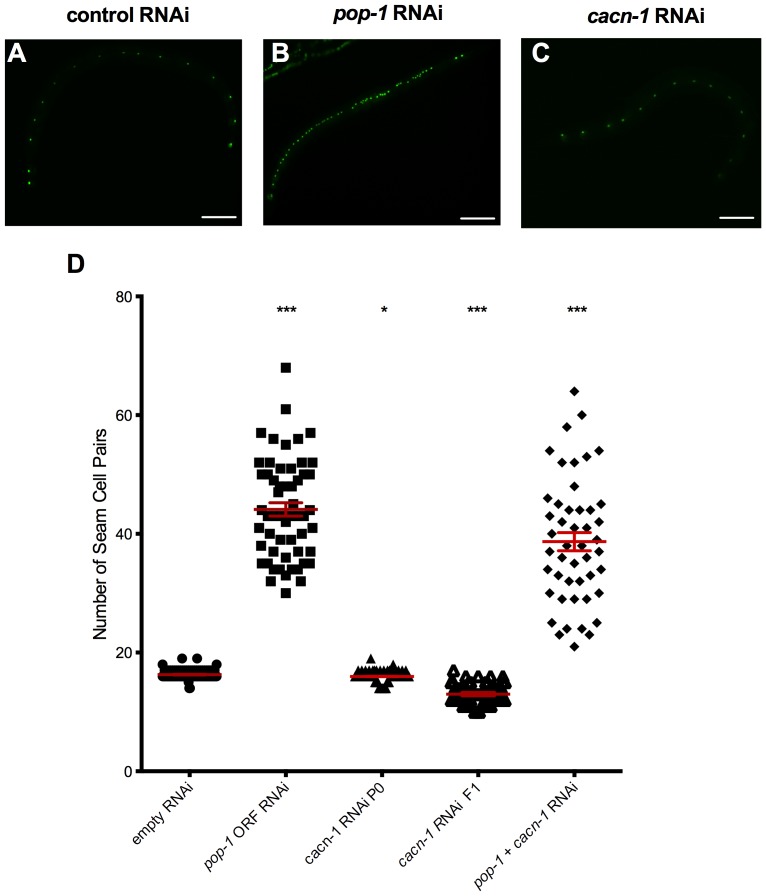
CACN-1 is required for normal seam cell proliferation. Representative fluorescence images of seam cells in *scm-1*p::GFP animals grown on *E. coli* HT115(DE3) carrying the (A) control RNAi, (B) *pop-1* ORF RNAi or (C) *cacn-1* RNAi vector. (D) *scm-1*p::GFP animals were grown on *E. coli* HT115(DE3) carrying empty vector, *pop-1* ORF RNAi, *cacn-1* RNAi or *cacn-1*+*pop-1* double RNAi vector. Individual points represent the number of seam cells for a single animal for a given RNAi treatment. Error bars indicate mean ± standard error of the mean. *** indicates statistical significance of P<0.001, and ** indicates statistical significance of P = 0.0013 compared to control RNAi. Scale bar is 20 µm.

Seam cell specification occurs early in larval development. In our standard protocol, animals treated with *cacn-1* ORF RNAi reach adulthood but are sterile. On average, these P0 animals have little reduction in seam cell number (15.9±0.1, P = 0.0135, *n = 73*), likely because CACN-1 is not effectively depleted early in larval morphogenesis. Animals expressing a strong loss of function allele of *cacn-1(tm3042)* arrest in the first larval stage, before seam cell number can be scored [51]. Therefore, we adopted a modified RNAi protocol to target the early larval stages. When L4 animals are transferred to *cacn-1* RNAi, a small percentage (1–5% N>1000) of the F1 progeny hatch and develop sufficiently for the seam cells to be scored. A similar attenuated RNAi protocol allowed identification of *cacn-1* in a genome-wide RNAi screen for novel regulators of seam cell development. In this screen, *cacn-1* RNAi animals displayed a reduction in terminal seam cell number to 13 seam cells [65]. We confirmed the decreased seam cell number in *cacn-1* RNAi animals; as expected, animals treated with control RNAi showed an average of 16.3±0.06 (*n* = 112) seam cells per side (Fig. 8A), whereas *cacn-1* RNAi animals had an average of 13.0±0.2 seam cells (P<0.0001, *n* = 65; Fig. 8C, D). Consistent with a previous report [34], we found that targeting *pop-1* with an RNAi construct corresponding to the entire *pop-1* ORF resulted in dramatic seam cell hyperplasia (44±1.12 seam cells per side, P = <0.0001, *n* = 56; Fig. 8B, D). In order to determine seam cell number in animals in which the expression of both *pop-1* and *cacn-1* is reduced, a double RNAi vector was prepared. This vector does not contain the entire *pop-1* ORF, therefore, we confirmed that depletion of *pop-1* using this targeting sequence does result in a significant increase in seam cell number (35±1.23, P<0.001, *n = 20*). Depleting both *pop-1*+*cacn-1* by double RNAi resulted in an increased number of seam cells that did not differ significantly from *pop-1* RNAi alone (38.7±1.54, P = 0.15, *n* = 47, Fig. 8D). These results suggest CACN-1 may work through POP-1 to exert its effects on seam cell proliferation.

**Figure 9:**
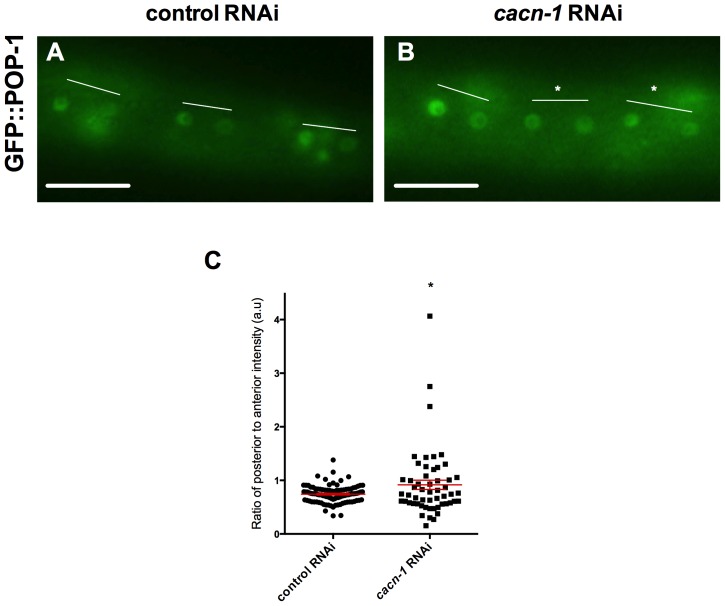
*cacn-1* RNAi alters the asymmetrical localization of GFP::POP-1 in seam cells. *sys-1*p::GFP::POP-1 animals were grown on *E. coli* HT115(DE3) carrying (A) control RNAi or (B) *cacn-1* RNAi vector. White lines indicate V3.pp(a/p), V4.pa(a/p) and V4.pp(a/p) seam cell daughters. In *cacn-1* animals the asterisks indicate equal POP-1 in both daughter cells of V4.pa(a/p) and V4.pp(a/p) divisions. Scale bar is 20 µm. (C) Individual points represent the posterior/anterior ratio of GFP expression in each seam cell pair for a given RNAi treatment. * indicates statistical significance of P = 0.011.

We predicted that CACN-1 would also influence POP-1 localization in seam daughters, similar to its role in the somatic gonad. To test this we used the GFP::POP-1 reporter, which displays asymmetric localization in wild type animals, with higher levels of POP-1 in the anterior daughter nucleus compared to the posterior daughter nucleus just after an asymmetrical division [30] (Fig. 9A). Qualitative assessment of the POP-1 distribution suggested that *cacn-1* RNAi could result in a loss of asymmetry, or even in a reversed polarity with higher levels of GFP::POP-1 in the posterior daughter nuclei (Fig. 9B). To further investigate the possibility of polarity reversal, we quantified the pixel intensity of each nucleus and calculated the posterior/anterior (reversed polarity) ratio of GFP::POP-1 for each seam cell pair (Fig. 9C). POP-1 in *cacn-1* RNAi treated seam cells was on average more evenly distributed, with some cells with reversed polarity, (0.92±0.087, *n* = 54) and differed significantly from control RNAi treated animals (0.74±0.16, P = 0.01, *n* = 98) in which the anterior cell expressed higher levels of GFP::POP-1. This result supports the conclusion that *cacn-1* RNAi can result in defective POP-1 asymmetry in dividing seam cells. Defects in POP-1 asymmetry may lead both daughter cells to adopt the anterior fate, contributing to the observed reduction in seam cell number in *cacn-1* RNAi treated animals.

## Discussion

Cactin is a novel protein linked to the regulation of several developmental signaling pathways. Here we reveal a novel potential role for the *C. elegans* Cactin homolog, CACN-1, in the Wnt signaling pathway. Depletion of CACN-1 by RNAi leads to an increase in POPTOP activation in the developing uterus and spermatheca of L4 animals. This rise in activation requires the activating β-catenin SYS-1 and correlates with an increase in POP-1 nuclear localization and a decrease in LIT-1, suggesting CACN-1 may exert its effects on POPTOP at least partially through LIT-1.

POP-1 is a transcription factor that regulates many cell fate decisions [18]–[22]. POP-1 usually acts to repress gene expression, unless bound to an activating β-catenin [13], [23]–[26]. Few POP-1 gene targets are known, most of which have expression limited to embryogenesis or early larval development [26], [66]–[69], therefore, in this study we primarily use POPTOP [38] as a proxy for regulation of gene expression by POP-1. POP-1 is regulated by several divergent β-catenins, SYS-1, WRM-1 and BAR-1 [61]. SYS-1 and BAR-1 are POP-1 co-activators [61]. Our results suggest SYS-1 is needed for POP-1 to activate POPTOP expression in the somatic gonad. We also observed that depletion of WRM-1 resulted in decreased expression of POPTOP. WRM-1 is a negative regulator of POP-1 and acts as an adaptor between POP-1 and LIT-1/NLK which marks POP-1 for phosphorylation and export [31], [61]. Without WRM-1, POP-1 may not be efficiently exported from the nucleus, perhaps leading to an increase in repressive POP-1 in the nucleus [61].

In the L4 uterine cells, we find high POPTOP expression correlates with high nuclear POP-1, which is contrary to the dominant paradigm that a low POP-1/SYS-1 ratio is needed for gene activation. One possibility is that SYS-1 levels are also increased by *cacn-1* RNAi. However, because available SYS-1 reporter constructs are not strongly expressed in the L4 somatic gonad (data not shown), we have not been able to directly test this idea. Perhaps more likely, POPTOP is an artificial construct that may not respond to POP-1/SYS-1 levels identically to promoters of endogenous target genes. POPTOP consists of seven POP-1 binding sites and the minimal *pes-1* promoter, and is maintained as a multi-copy transgene. We suggest POPTOP acts as a “POP-1 sponge”, requiring higher levels of POP-1 than normal for activation. In addition, a recent report shows that a second binding site, the HMG-helper site, is needed for robust expression of a variety of different Wnt-responsive genes. Addition of HMG-helper sites to a POP-1-responsive reporter construct (POPHHOP) results in stronger POP-1 binding and higher expression levels [70]. These two factors, copy number and lack of a helper site, may help to explain why POPTOP has very low basal levels of expression, and is expressed only in a subset of the cells known to be responsive to Wnt signaling.

Although the overall expression pattern does differ between the two constructs [70], POPHHOP and POPTOP are both expressed at low levels in the spermatheca and in cells surrounding the vulva in L4 animals (Fig. S1). We find that POPHHOP responds to *cacn-1* RNAi with an increase in the number of nuclei expressing POPHHOP::GFP and an increase in the average fluorescence intensity (Fig. S1). The increase in POPHHOP expression in response to *cacn-1* RNAi is not as striking as seen in POPTOP (Fig. 2) perhaps due to higher levels of basal expression in the less-repressed POPHHOP construct, or to other differences in the promoter and 3′UTR of the two constructs. We propose that when *cacn-1* is depleted, POP-1 then accumulates to sufficient levels to activate POPTOP (and POPHHOP), suggesting CACN-1 normally acts as a negative regulator of POP-1. This is consistent with our findings in larval seam cells, where CACN-1 appears to act through POP-1 to support normal levels of seam proliferation.

Depletion of CACN-1 also results in a decrease in LIT-1 expression. LIT-1 activity is required for many POP-1-dependent events during *C. elegans* development, such as asymmetric cell division in embryos [71] and proximal-distal axis determination during larval gonad development [30]. Because LIT-1 is needed to phosphorylate POP-1 and mark it for nuclear export, the accumulation of POP-1 in the nucleus of *cacn-1* depleted animals may be a direct result of a loss of LIT-1. However, the additive effect of loss of *cacn-1* and *lit-1* on POPTOP expression suggests that CACN-1 may also function in parallel to LIT-1 (Fig. 5). These results suggest CACN-1 negatively regulates POP-1 by targeting both POP-1 and LIT-1 expression and/or localization.

We have demonstrated that CACN-1 plays a role in seam cell development. The role of the Wnt pathway in seam cell specification is complex and involves timing in the seam lineages [72]. This critical timing element, combined with the small percentage of surviving animals in our *cacn-1* RNAi experiments, makes an RNAi approach to studying the seam cell lineage problematic. Identification of a conditional allele of *cacn-1* would allow the role of CACN-1 in seam cell specification to be explored through lineage analysis and through genetic interaction studies. For example, when in development is CACN-1 needed? Does CACN-1 play a role in lineage commitment in the seam cells or in the somatic gonad? What is the effect of combining *cacn-1(RNAi)* with loss of *lit-1* or other Wnt-pathway genes? Exploring these questions, and others, would help to elucidate the role CACN-1 may play in Wnt/asymmetry pathway signaling and nematode development.

CACN-1/Cactin is a predominantly nuclear, coiled coil domain containing protein of unknown molecular function and no obvious nucleic acid binding or enzymatic activity [42], [51]. Short coiled coil domains serve as sites of homo- and hetero-dimerization and can act as scaffolds for modulating protein interactions [73]. Evidence is accumulating that Cactin may play a role in RNA splicing or post-transcriptional gene regulation. In *Arabidopsis*, CACTIN co-localizes with spliceosomal proteins in nuclear speckles [46] and interacts with spliceosomal components in Drosophila and human cells [47], [74]. In the *C. elegans* germline, loss of CACN-1 (W03H9.4) leads to similar phenotypes as depletion of other splicing factors [48], [75]. Therefore, our working model is that CACN-1 functions in the nucleus primarily through scaffolding the protein-protein interactions needed for proper processing, localization or efficient translation of mRNAs. In this role, CACN-1 might affect the levels or isoform expression of stage- or cell-type specific regulatory genes. It is possible that CACN-1 may be directly required for proper expression or localization of LIT-1 and POP-1 in the somatic gonad and seam cells, or the effect of CACN-1 could be fairly indirect. For example, depletion of CACN-1 could have a general effect on proper cell lineage commitment, leading to the observed inappropriate distribution of POP-1 and LIT-1 in these cells. Given that the *cacn-1* null allele *tm3042* arrests in L1 [51], *cacn-1* may broadly affect developmental gene regulation, including Wnt regulated processes.

In summary, this study identifies a potential role for the well-conserved nuclear protein Cactin in Wnt/asymmetry pathway signaling. Proper regulation of TCF/LEF is critical for normal development in all animals [76] and improper regulation of Wnt signaling can promote metastatic behavior of cells [31], [32], [77]–[79], therefore, further studies investigating how Cactin regulates TCF/LEF in other animals and in clinical contexts may reveal new insights into basic developmental mechanisms and eventually lead to new therapeutic strategies.

## Supporting Information

Figure S1 **CACN-1 is a negative regulator of POPHHOP reporter expression.** The POP-1 and HMG-helper optimal promoter (POPHHOP) reporter animals express a construct consisting of six HMG-helper sites driving GFP. The HMG-helper site supports higher expression levels in a variety of Wnt-responsive promoter contexts and cell types [70]. POPHHOP animals were grown on *E. coli* HT115(DE3) carrying the (A) control RNAi vector or (B) *cacn-1* RNAi vector for 44 hours at 23°C. Asterisks indicate the L4 developing vulva. Control animals most commonly express GFP brightly in two cells flanking, and slightly above, the developing vulva. *cacn-1* RNAi animals express POPHHOP throughout the somatic gonad. (C) Individual points represent the number of POPHHOP expressing nuclei in the somatic gonad for a single animal for a given RNAi treatment. (D) Individual points represent total pixel intensity (a.u.) for a single animal for a given RNAi treatment. Data were analyzed as described for POPTOP in the materials and methods. Because these data are not normally distributed, the error bars indicate the median and interquartile range of the data set, and the non-parametric Kologorov-Smirnov test was used to compare the populations. * indicates statistical significance of P = 0.01, ** indicates statistical significance of P<0.01. Scale bar is 25 µm.
(TIFF)
