# Supplementary figures and images for: CACN-1/Cactin Plays a Role in Wnt Signaling in C. elegans
Source: PLoS One. 2014 Jul 7;9(7):e101945. doi: 10.1371/journal.pone.0101945 (PMC4084952; doi:10.1371/journal.pone.0101945)

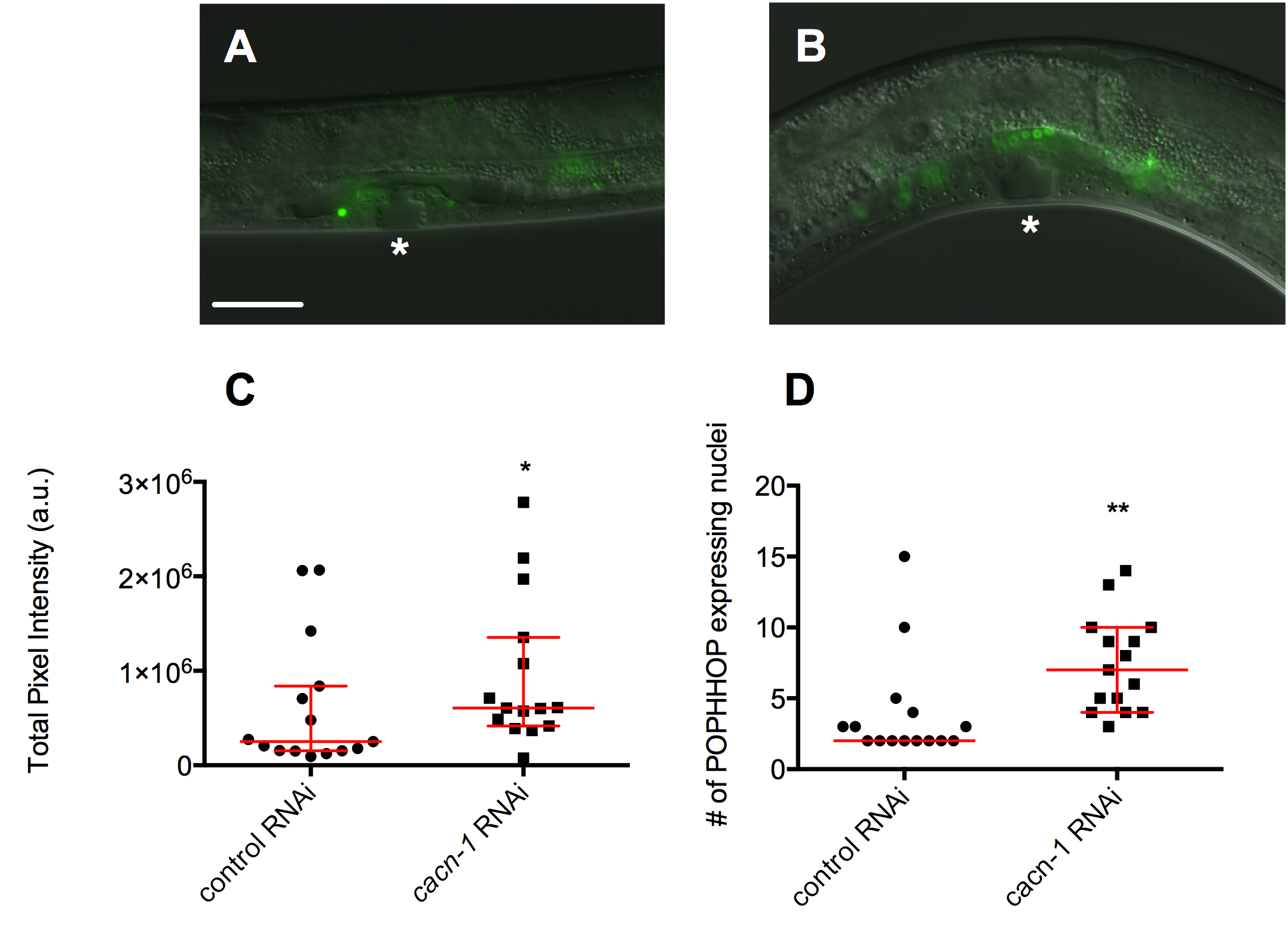

Supplement: Figure S1 — CACN-1 is a negative regulator of POPHHOP reporter expression. The POP-1 and HMG-helper optimal promoter (POPHHOP) reporter animals express a construct consisting of six HMG-helper sites driving GFP. The HMG-helper site supports higher expression levels in a variety of Wnt-responsive promoter contexts and cell types [70]. POPHHOP animals were grown on E. coli HT115(DE3) carrying the (A) control RNAi vector or (B) cacn-1 RNAi vector for 44 hours at 23°C. Asterisks indicate the L4 developing vulva. Control animals most commonly express GFP brightly in two cells flanking, and slightly above, the developing vulva. cacn-1 RNAi animals express POPHHOP throughout the somatic gonad. (C) Individual points represent the number of POPHHOP expressing nuclei in the somatic gonad for a single animal for a given RNAi treatment. (D) Individual points represent total pixel intensity (a.u.) for a single animal for a given RNAi treatment. Data were analyzed as described for POPTOP in the materials and methods. Because these data are not normally distributed, the error bars indicate the median and interquartile range of the data set, and the non-parametric Kologorov-Smirnov test was used to compare the populations. * indicates statistical significance of P = 0.01, ** indicates statistical significance of P<0.01. Scale bar is 25 µm. (TIFF) [file pone.0101945.s001.tiff]
